# Informing Your Future Clozapine Patients of the Mortality Risk: Fear of Agranulocytosis vs. Pharmacovigilance Fatal Outcomes

**DOI:** 10.62641/aep.2486

**Published:** 2026-10-15

**Authors:** Jose de Leon, Can-Jun Ruan, Georgios Schoretsanitis, Emilio J. Sanz, Carlos De las Cuevas

**Affiliations:** ^1^Mental Health Research Center at Eastern State Hospital, Lexington, KY 40511, USA; ^2^Biomedical Research Centre in Mental Health Net (CIBERSAM), Santiago Apóstol Hospital, University of the Basque Country, 01006 Vitoria, Spain; ^3^The National Clinical Research Center for Mental Disorders and Beijing Key Laboratory of Mental Disorders, Beijing Anding Hospital, Capital Medical University, 100088 Beijing, China; ^4^Advanced Innovation Center for Human Brain Protection, Capital Medical University, 100088 Beijing, China; ^5^Department of Psychiatry, Lausanne University Hospital and University of Lausanne, CH-1008 Prilly-Lausanne, Switzerland; ^6^Unit of Pharmacogenetics and Clinical Psychopharmacology, Center for Psychiatric Neurosciences, Lausanne University Hospital and University of Lausanne, CH-1005 Lausanne, Switzerland; ^7^The Zucker Hillside Hospital, Psychiatry Research, Northwell Health, Glen Oaks, NY 11004, USA; ^8^Department of Psychiatry, Zucker School of Medicine at Northwell/Hofstra, Hempstead, NY 11549, USA; ^9^Department of Physical Medicine and Pharmacology, School of Medicine, University of La Laguna, 38200 Tenerife, Canary Islands, Spain; ^10^Hospital Universitario de Canarias, 38320 Tenerife, Spain; ^11^Department of Internal Medicine, Dermatology and Psychiatry, School of Medicine, and Instituto Universitario de Neurociencia (IUNE), University of La Laguna, 38200 Tenerife, Canary Islands, Spain

## Introduction

Schizophrenia is associated with substantial loss of years of life [1]. Three major reasons explain this increase in deaths: (a) a 5% lifetime risk of dying by suicide [2]; (b) very poor adherence, particularly ≥ one year after psychotic exacerbations [3]; and (c) the strong association with smoking and heavy smoking [4], leading to higher risk of cancer and cardiovascular deaths. Treatment-resistant schizophrenia (TRS) is likely to be even more lethal, but it has never been studied in epidemiological studies.

Clozapine can reduce this increase in fatal outcomes in schizophrenia due to its: (a) anti-suicide effects [5], (b) superior adherence among the oral antipsychotics [6], and (c) positive effects on comorbid substance abuse [7], although its effects on tobacco smoking are less clear. Clozapine is the only antipsychotic approved by the Food and Drug Administration (FDA) for suicide prevention and according to the United States (US) studies and the Finnish registry, it may prevent up to 80% of suicide deaths [5]. The Scandinavian registries indicate that clozapine is not only the best oral antipsychotic regarding adherence, but clozapine treatment may be associated with the best adherence among medications that treat cardiovascular disease [8].

By stressing agranulocytosis too much, the FDA has indirectly promoted clozaphobia [8]; however, under hematological monitoring agranulocytosis mortality during clozapine treatment is negligible. In a 19 November 2024 meeting [8], the FDA described a fatal outcome rate of 0.03% (2 deaths in 6488 veteran patients followed for up to 23 years). Since clozapine’s introduction in Japan there have been 2 deaths, which produced a fatal outcome rate of 0.008% (2/25,214) [9]. Its complex pharmacokinetics and lack of dosing studies [6] contributed to clozapine’s reputation as a “toxic” drug. This can be fixed first by using personalized titrations with C-reactive protein (CRP) monitoring to avoid clozapine-induced inflammation manifested as fever or even myocarditis [10]. Second, much lower maintenance doses are required, particularly in patients whose ancestry is Asian (specifically originating geographically between Pakistan and Japan) or are descendants of the original inhabitants of the Americas, who require half of the US-approved dosing levels [6]. Moreover, maintenance doses close to the minimum therapeutic dose (MTD) (providing 350 ng/mL of serum clozapine concentration) may help avoid clozapine intoxication after the development of a pneumonia; the top fatal outcome of clozapine-treated patients [8].

FDA regulations dictate that boxed warnings need to reflect fatal outcomes found during postmarketing surveillance [6]. We studied 22,908 deaths occurring worldwide until the beginning of 2023 [11], including 17,624 identified as adults in VigiBase, the international pharmacovigilance database (footnote a Table 1). Pneumonia was the top specific fatal outcome in VigiBase and the top burden in the Finnish registry [12].

**Table 1.** **VigiBase adult fatal outcomes (early 2023) and burden in Finland**.

Fatal outcome label/group		VigiBase adult fatal outcomes		Finnish study		Likely contributor
		All 17,624 (%)^a^	Specific 11,849 (%)^b^	2659 patients	Cumulative incidence at 20 years		
Single label						
	Agranulocytosis	189 (1.1%)	1.6%	58 (2.1%) agranulocytosis <5 fatal outcomes (<0.2% of 2659)	2.4%	CLO
	Myocarditis	93 (0.5%)	0.8%	<5 (<0.2%)	Not calculated	CLO
	Pneumonia	1562 (8.9%)	13.2%	408 (15.3%) at least 1 episode 24 fatal outcomes (0.9% of 2659)	29.5%	CLO & TRS
	Myocardial infarction	990 (5.6%)	8.4%	Not studied	Not studied	TRS
	Completed suicide	617 (3.5%)	5.2%	Not studied	Not studied	TRS
	Lung cancer	436 (2.5%)	3.7%	Not studied	Not studied	TRS
	Pulmonary embolism	444 (2.5%)	3.7%	31 (1.2%) pulmonary embolism 49 (1.8%) VTE	Not calculated	TRS
Combined labels						
	Respiratory complications^c^	2270 (12.9%)	19.2%	Unclear whether or not they overlap with pneumonia 201 (7.6%) acute upper respiratory 151 (5.7%) other lower respiratory 137 (5.2%) bronchitis 69 (2.6%) influenza 26 (1.0%) aspiration pneumonia	17.6% 10.4% Not calculated Not calculated Not calculated	CLO & *TRS*^d^ Aspiration pneumonia caused by CLO and co-medications Infectious pneumonia in the absence of aspiration mainly due to TRS
	Acute cardiac fatal outcomes^e^	2289 (13.0%)	19.3%	Not studied	Not studied	TRS & *CLO*^f^ Myocardial infarction mainly due to TRS CLO can cause TdP
	All suicide labels^g^	734 (4.2%)	6.2%	Not studied	Not studied	TRS
	All cancer labels	785 (4.5%)	6.6%	Not studied	Not studied	TRS
	All other infections (leukocytosis^h^ in VigiBase)	1088 (6.2%)	9.2%	133 (5.0%) bacterial infections 131 (4.9%) intestinal infections 115 (4.3%) pyelonephritis	8.5% 10.0% Not calculated	TRS
	Estimated^i^ CIGH	636 (3.6%)	5.4%	338 (12.7%) constipation 79 (3.0%) ileus (12 fatal 0.5% of 2659)	22.7% 5.3%	CLO &TRS
	All CLO-induced inflammations	Not studied		35 (1.3%) local inflammation^j^ 151 (5.7%) benign fever	Not calculated Not calculated	CLO

CIGH, clozapine-induced gastrointestinal hypomotility; CLO, clozapine; TRS, treatment-resistant schizophrenia; VTE, venous thromboembolism; TdP, torsades de pointes. ^a^Of 22,908 fatal outcomes in early 2023, 42 patients <18 years old and 5284 with age missing were eliminated to lead to these 17,624 adult fatal outcomes. ^b^Of the 17,624 adult fatal outcomes, there were 5775 that were described only with the label “death”; therefore we have no specific information on them. The other 11,849 were considered to have a specific fatal outcome when at least one specific ADR label was listed. Thus, the percentage of this specific fatal outcome was calculated. It is possible that this percentage better reflects clinical relevance, as some of the cases of each ADR potentially occurred but were not identified in the patients classified only with the label “death”. ^c^These respiratory complications likely associated with swallowing impairment combined four labels: pneumonia, aspiration pneumonia, lower respiratory tract infection, and chronic obstructive pulmonary disease, after eliminating overlaps. ^d^The smaller italic font for TRS indicates that it has less influence than CLO. Most of the respiratory complications in the spectrum of aspiration pneumonia are probably explained by CLO and other drugs that interfere with swallowing. Less frequently, patients had infectious pneumonia in the absence of aspiration, which may be more associated with TRS and smoking. ^e^They combined three labels: myocardial infarction, cardiac arrest and sudden death after eliminating overlaps. ^f^The smaller italic font for CLO indicates that it has less influence than TRS. Most of the acute cardiac outcomes are probably explained by TRS. Less frequently, patients may have died of torsades de pointes which may be associated with high doses of CLO. ^g^Some deaths are not reported as completed suicide but as attempted suicide with fatal outcome or intentional overdose with fatal outcome. Based on our prior article focused on suicide, we estimate them to be 1.19 times higher (617 × 1.19 = 734). ^h^Includes fatal outcomes in VigiBase from 3 labels for leukocytosis (increased neutrophils, increased white blood cells and neutrophilia) after eliminating overlaps; most fatal outcomes from these 3 ADR labels were associated with infections. However, many fatal outcomes during infections may not be reported as leukocytosis but across >120 labels for different infections; thus, as far as we can tell, leukocytosis provides a major underreport of infections other than pneumonia. To study all fatal outcomes in VigiBase associated with all infections would require studying >120 ADR labels. ^i^In early 2026, the number of fatal outcomes was 28,645 or 1.25 times higher than in early 2023 (28,645/22,956 = 1.25). In the beginning of 2026, there were 795 fatal outcomes in adults associated with any of the 11 CIGH labels after correcting for overlaps. To estimate the fatal outcomes in early 2023, we divided them by 1.25 (795/1.25 = 636). ^j^They described 13 cases of colitis, 10 of interstitial nephritis, 8 of pancreatitis and <5 of myocarditis/pericarditis. These 35 cases of clozapine-induced inflammation assume no overlap among these types of inflammation. Overlap in a few cases is expected but the authors of the Finnish study provided no data on the overlap in the article or when requested by the first author of this editorial.

When a clozapine-treated patient dies, the cause of death could be associated with: (a) a clozapine adverse drug reaction (ADR), (b) TRS or (c) the combination [13]. This is extremely important if you are discussing with patients and families the risks/benefits of starting clozapine; they need to consider the risk of dying from TRS, particularly suicide, when not using clozapine [5].

A prior editorial [13] compared the risk of dying of agranulocytosis against the four top labels in VigiBase after age stratification. This analysis expands that editorial by providing a summary of all fatal outcomes in clozapine-treated patients in VigiBase, interpreting them as caused by clozapine, TRS or the combination. This data is compared with the data from the Finnish registry [12].

## Methods

VigiBase is the World Health Organization’s pharmacovigilance database of potential ADRs from medicinal products, collected from all national drug/pharmacovigilance agencies. The reporting agent sometimes classifies ADRs, but usually those who report free text information and the pharmacovigilance staff at a regional/national drug agency or pharmaceutical company encode them, using VigiBase’s labels. Of the 22,908 fatal outcomes [11] we focused on 17,624 in adults from onset through 15 January 2023 [14]. Reports of fatal outcomes in VigiBase frequently include several labels, thus, to better understand them requires studying all fatal outcomes among overlapping labels. In Finland, clozapine-induced gastrointestinal hypomotility (CIGH) was listed as the second cause of burden but in VigiBase CIGH is reported using many labels [8]. All VigiBase reports from 11 labels possibly associated with CIGH until 27 January 2026 were studied.

## Results

Table 1 summarizes the most important fatal outcomes in VigiBase and uses Finnish data as a comparison. VigiBase is contaminated by agranulocytosis deaths before hematological monitoring was required. The agranulocytosis label explained only 1.1% of the deaths. In the 2659 Finnish patients, less than 5 patients died of agranulocytosis, which provided <0.2% of the fatal outcomes.

Pneumonia was the top fatal outcome in VigiBase and the top burden in Finland. In VigiBase when a broader category was used, “respiratory complications likely associated with swallowing impairment” (footnote b of Table 1), it explained 12.9% of fatal outcomes worldwide.

Myocardial infarction accounted for 5.6% of VigiBase fatal outcomes but was not studied in Finland. When combined with cardiac arrest and sudden death (footnote d of Table 1), they explained 13.0%.

The third large category is probably other infections, represented in VigiBase as producing 6.2% of fatal outcomes in the presence of leukocytosis. The Finnish study reported a large burden for infections other than pneumonia, but this was not commented on in the published article. Other VigiBase important fatal outcomes were all cancer labels (4.5%), all suicide labels (4.2%), estimated CIGH (3.6%) and pulmonary embolism (2.5%).

## Discussion

Next, we discuss how to inform patients and families, both in general and after considering that VigiBase fatal outcome rates in clozapine-treated patients vary with age.

### Informing Your Patient of the Risk of Fatal Outcomes Associated With TRS

Any discussion with a patient and his/her family considering clozapine needs to acknowledge that TRS is associated with fatal outcomes, including a 5% lifetime risk of death by suicide [2] and a high risk of death from infection or a cardiovascular cause [1].

### Informing Your Patient of the Extremely Low Risk of Fatal Outcomes Associated With Agranulocytosis

If hematological monitoring is used, recent studies indicate that agranulocytosis has <1% risk of mortality. In the US, in July 2025, the FDA did not change the frequency of monitoring of absolute neutrophil counts but relabeled it as “recommended”. Thus, your patient and his/her family need to be informed that the literature exaggerates the risk of fatal outcomes in agranulocytosis and, when preferred by the patient, you should discuss off-label options of less burdensome hematological monitoring.

### Informing Your Patient of the Risk of Fatal Outcomes Associated With CIGH

All patients may need to be informed that constipation can lead to fatal outcomes and that prophylaxis with laxatives and monitoring bowel movements may reduce mortality across all ages.

### Informing Your Patient of the Risk of Fatal Outcomes Associated With Pneumonia and Other Infections

The major risk of death for a clozapine-treated patient is not from agranulocytosis but from pneumonia and other severe infections. Infection releases cytokines which can cause clozapine intoxication; thus, the combination is very lethal. Pneumonia mortality varies with age, sex and duration of treatment (0–76%) [14]. It is very important to educate patients and family to contact you when any signs of infection, including fever, are present. If CRP is elevated and clozapine levels are not immediately available, it may be safer to halve the clozapine dose [14].

### Informing Your Geriatric (≥65 Years) Patient of the Risk of the Foremost Fatal Outcome: Aspiration Pneumonia

In VigiBase, geriatric patients account for 30% of fatal outcomes; these are patients who have not already died from suicide, infections, cardiovascular death, or cancer. Up to 30% of fatal outcomes are from aspiration pneumonia, caused by clozapine and the co-medications that interfere with swallowing (benzodiazepines, olanzapine, risperidone and valproate); thus, if the patient survives, dose reduction to the MTD and stopping other co-medications that interfere with swallowing should be considered [14]. The US package insert does not state that clozapine metabolites are mainly eliminated by the kidneys, and as a patient reaches geriatric age, dose reduction by approximately 30% should be considered to compensate for the decrease in renal clearance. Thus, as patients reach the 6th decade, dose reductions based on clozapine levels should be considered [7].

### Informing Your Middle-Aged (45–64 Years) Patient of the Risk of the Foremost Fatal Outcome: Pneumonia and Other Infections

In VigiBase, middle-aged clozapine-treated patients accounted for 45% of fatal outcomes. The top fatal outcome was pneumonia which can be (a) aspiration pneumonia or (b) infectious pneumonia in the absence of swallowing impairment. Pneumonia in the absence of swallowing impairment and other infections may be more reflective of TRS than clozapine.

Myocardial infarction is an important fatal outcome in middle-aged patients. The literature and a VigiBase comparison with other antipsychotics suggest that myocardial infarction is likely associated with TRS [15].

### Informing Your Young (18–44 Years) Male Patient of the Risk of the Foremost Fatal Outcome: Suicide

In VigiBase, young males account for 18% of fatal outcomes. The most important cause is suicide with 9.2% fatal outcomes [14]. Taking clozapine consistently may reduce deaths by 80%; thus, if the young male patient considers taking clozapine, you need to discuss using clozapine levels to monitor adherence. If he refuses to consider clozapine, you need to encourage a long-acting antipsychotic [5].

Similarly, with 26% fatal outcome, suicide was the top cause of death in adolescent clozapine-treated patients [14]. The same discussion may be required in young females, as suicide is the second leading cause of fatal outcomes.

### Informing Your Young Female Patient of the Risk of the Foremost Fatal Outcome: Pulmonary Embolism

In VigiBase, young females account for only 7% of fatal outcomes. For them, pulmonary embolism is the top fatal outcome with 8.0% of fatal outcomes [14]; thus, it may be important to discuss obesity and limited mobility as risk factors for pulmonary embolism. Inform the patient that if she is taking oral contraceptives, they increase the risk of pulmonary embolism, and are inhibitors of clozapine metabolism, so these patients may need half of the clozapine dosage or, better yet, consider MTD using clozapine levels.

### Limitations

The limitations of these pharmacovigilance studies are described in prior articles [8, 11]. Four countries (the US, the United Kingdom, Canada and Australia) with registries provide most (93%) of the VigiBase fatal outcome reports; it is not known whether other areas of the world have the same or different profiles for fatal outcomes in clozapine-treated patients.

## Conclusions

We propose two new ideas. First, any discussion of the risks/benefits of starting clozapine or not needs to consider the lethality of TRS and that 5% of patients with schizophrenia may die by suicide; consistent adherence to clozapine may cut these deaths by 80%. Second, the patient and family need to be informed that with hematological monitoring the risk of death by agranulocytosis is extremely low (<1%), however, they need to cooperate with you to early diagnose and treat pneumonia and other infections, the major cause of fatal outcomes in clozapine-treated patients.

## Availability of Data and Materials

VigiBase does not allow distribution of their data, but many more details are provided in previously published articles.
